# The Effect of GBFS on the Mechanical Properties and Hydration Products of Steam-Cured Cement Mortar

**DOI:** 10.3390/gels12020110

**Published:** 2026-01-27

**Authors:** Baoliang Li, Jie Li, Yue Li, Hongrui Shang, Haohang Yu, Binbin Huo, Yuyi Liu

**Affiliations:** 1 Faculty of Architecture and Civil Engineering, Huaiyin Institute of Technology, Huaian 223001, China; 18651985707@163.com (J.L.); shang_hongrui@126.com (H.S.); sr_yuhaohang@126.com (H.Y.); liuyuyi88@163.com (Y.L.); 2 Jiangsu Key Laboratory of Construction Materials, School of Materials Science and Engineering, Southeast University, Nanjing 211189, China; huobinbin@cumt.edu.cn; 3 School of Mines, China University of Mining and Technology, Xuzhou 221116, China

**Keywords:** GBFS, steam-cured cement mortar, mechanical properties, hydration products

## Abstract

To investigate the mechanism by which ground granulated blast-furnace slag (GBFS) affects the performance of steam-cured cementitious materials, this study systematically analyzes the effect of GBFS on the mechanical strength and hydration products of mortar by adjusting the GBFS content (0%, 20%, 30%, 50%), curing temperature (50 °C for 7 h, 80 °C for 7 h), and curing time (3 d, 28 d). The results show that although increasing the steam-curing temperature can improve the strength activity index of GBFS-containing mortar, higher temperatures tend to induce later-age strength retrogression in such mixtures. Steam-curing not only promotes the massive formation of calcium hydroxide with coarse crystals but also increases the initial Ca/Si ratio of calcium silicate hydrate (C–S–H) gels, which is a crucial factor contributing to the high susceptibility of steam-cured concrete to brittle fracture; however, the incorporation of GBFS can effectively mitigate this issue. Furthermore, under the steam-curing condition of 80 °C, the addition of GBFS facilitates the formation of hydrogarnet and delayed ettringite, which is unfavorable for the long-term strength development and durability improvement in concrete.

## 1. Introduction

Concrete, as the most widely used construction material in the world today, has always been a focal point of research for optimizing its construction efficiency and service performance [1]. Steam-curing technology, which accelerates the hydration process of concrete by artificially controlling temperature and humidity conditions, significantly shortens the curing cycle and enhances construction efficiency [2]. It has thus become one of the main curing methods for the production of precast concrete components. Compared with natural curing, steam-curing can not only greatly shorten the construction period but also improve the early strength and structural compactness of concrete under specific conditions, making it one of the key technologies driving the development of construction industrialization [1,2].

With the in-depth implementation of the green building material concept and the continuous rise in demand for resource recycling, the resource utilization of industrial solid waste has become a crucial development direction in the field of concrete materials. Granulated blast-furnace slag (GBFS) is a by-product of the ironmaking industry, produced through high-temperature melting followed by water quenching. Owing to its abundant amorphous phases, it possesses both latent hydraulicity and pozzolanic activity and is thus commonly ground into fine powder to serve as a mineral admixture for concrete [3,4]. The chemical composition of GBFS powder is dominated by CaO, SiO_2_, Al_2_O_3_, and MgO [5]. When replacing a portion of cement in concrete production, GBFS not only reduces cement dosage, cuts carbon emissions and resource consumption [6], but also optimizes the internal structure of concrete via its filling and pozzolanic effects, thereby enhancing the later-age strength [7] and durability of concrete, including impermeability [8], sulfate attack resistance [9,10], frost resistance [11], and high-temperature performance [12]. Therefore, investigating the mechanism of GBFS in steam-cured concrete is of significant importance for promoting the greening and high performance of concrete materials. Although domestic and international scholars have conducted extensive research on the effects of GBFS as an admixture on concrete performance, studies specifically addressing its influence on the hydration products, microstructure, and mechanical properties evolution of concrete under steam-curing conditions remain relatively scarce.

The key factor determining whether GBFS can be applied to steam-cured concrete components depends on its intrinsic reactivity level. The reactivity of GBFS powder is primarily influenced by chemical composition, amorphous phase content, fineness, as well as the alkalinity and temperature of the hydration environment [13]. Specifically, the reactivity of GBFS is directly proportional to its amorphous phase content and increases with higher fineness [14]. Meanwhile, the chemical composition of GBFS (e.g., Al_2_O_3_, MgO, and CaO) serves as an inherent factor determining its hydration reactivity and the characteristics of hydration products [15]. Among these, the influence of Al_2_O_3_ content exhibits duality and interacts with other components: Wan et al. [16] indicated that when the Al_2_O_3_ content does not exceed 15%, Al_2_O_3_ has a positive effect on the reactivity of GBFS. However, due to the interaction between Al_2_O_3_ and CaO, its promoting effect also depends on the CaO content. This influence is significantly more pronounced in high-calcium GBFS compared to low-calcium GBFS. From the perspective of microstructural characteristics of hydration products, Haha et al. [15] found that an increase in Al_2_O_3_ content in GBFS reduces the Mg/Al ratio of hydrotalcite, increases the solid solution content of Al in calcium silicate hydrate (C–S–H), and promotes the formation of strätlingite. In addition, MgO content also plays a significant regulatory role in the hydration products and strength development of GBFS. Further research by Haha et al. [17] revealed that when the MgO content in GBFS increased from 8% to 13%, the generation of hydrotalcite in a water glass-activated GBFS paste system increased, while the adsorption capacity of Al by C–S–H decreased.

Compared to chemical composition, temperature exerts a more significant influence on the reactivity of GBFS, and this influence pattern differs remarkably from that observed in the pure Portland cement (PPC) system [14]. Wu et al. [18] have confirmed that at room temperature, GBFS undergoes distinct hydration within one day and exhibits higher sensitivity to temperature variations; at normal temperature (27 °C), the cumulative heat release of PPC within one day is 1.61 times that of GBFS-based cement, significantly exceeding the latter; conversely, when the temperature rises to 60 °C, this pattern reverses, with the cumulative heat release of GBFS-based cement reaching 1.35 times that of PPC within one day. Barnett et al. [19] also identified the same pattern under the conditions of 0%~70% GBFS content and a temperature range of 10~50 °C: the strength growth of GBFS-blended mortar under standard curing is slower than that of PPC, while it accelerates significantly at elevated temperatures; additionally, the higher the GBFS content, the more pronounced the early strength improvement. However, relatively high early-age curing temperatures are generally detrimental to the later-age strength development of cementitious materials. Çakır et al. [1,20] verified that temperature elevation within the range of 20~40 °C can enhance the early strength of mortar but reduce its later-age strength, and this effect is more prominent in GBFS-blended mortars compared with the plain group. Escalante et al. [21] carried out a six-month curing study on composite cement pastes with 30% and 50% GBFS content at temperatures ranging from 10 °C to 50 °C, and the results showed that the hydration reactivity of GBFS increases with rising curing temperatures, whereas it decreases with increasing GBFS content. Roy and Idorn [22] discovered that the alkaline components released during clinker hydration can effectively activate GBFS, and that the temperature rise in the initial stage of hydration can provide energy for the alkaline erosion reaction on GBFS particles. This activation mechanism also provides a reasonable explanation for the aforementioned research results of Escalante et al. regarding the effects of temperature and GBFS content on hydration reactivity, indicating that the hydration rate of GBFS in composite cement is ultimately determined by the synergistic effect of the thermal environment and alkaline environment regulated by curing conditions.

Increasing the curing temperature can shorten the construction timeline of concrete, but due to the rapid hydration of cement clinker and the uneven precipitation of hydration products, it introduces thermal damage, such as expansion and cracking, within the concrete [1,2]. However, GBFS is particularly helpful in mitigating the thermal damage in steam-cured concrete and compensating for later strength loss [3]. This is mainly related to the fact that, after early high-temperature curing, the later hydration of the cement component in the composite cementitious material is inhibited, while the later reaction of the GBFS component is less affected. The further reaction of GBFS increases the production of C–S–H gels, which improves the pore structure [23]. At the same time, under high-temperature curing conditions, GBFS can promote the hydration of various mineral phases in cement clinker and reduce the formation of calcium hydroxide (CH) in hardened cement paste [24]. However, compared to fly ash, GBFS has a relatively limited ability to consume CH in hardened cement paste under high-temperature curing [25]. Meanwhile, Zhuang [26] reported that GBFS can enhance the demolding strength of steam-cured concrete, improve its sulfate resistance, and reduce its permeability.

Although existing literature has explored the impact of elevated curing temperatures on the performance of GBFS-based cementitious materials, the high-temperature curing conditions described in these studies differ from those used in modern precast concrete elements. Currently, precast concrete elements are cured at higher temperatures, typically between 65 °C and 90 °C, with the high-temperature curing process predominantly completed within the first 24 h rather than involving prolonged exposure. Additionally, GBFS contains higher levels of Al_2_O_3_, SiO_2_, and SO_3_ compared to PPC. However, the specific effects of GBFS on the hydration products and microstructure of cement under the steam-curing conditions commonly applied in precast concrete production remain unclear.

To address this gap, this study focuses on the influence of GBFS on the hydration products and mechanical properties of steam-cured concrete at different temperatures. Specifically, a series of experiments with multi-gradient steam-curing temperatures (50 °C for 7 h and 80 °C for 7 h) and GBFS replacement levels (0%, 20%, 30%, and 50%) were designed to systematically analyze the microstructural characteristics of hydration products, hydration degree, and the variation laws of macroscopic mechanical properties. Through this systematic analysis, the study aims to reveal the coupling mechanism between curing temperature and GBFS replacement level. This research can not only enrich the hydration theory of steam-cured concrete containing GBFS but also provide a scientific basis for optimizing mix design and curing processes in practical engineering applications of steam-cured concrete.

## 2. Results and Discussion

### 2.1. Results of Material Characterization

The chemical compositions of ordinary Portland cement (OPC) and ground granulated blast-furnace slag (GBFS) measured by XRF are listed in Table 1. GBFS was primarily composed of CaO, SiO_2_, and Al_2_O_3_, with the contents of Al_2_O_3_, MgO, and SO_3_ significantly higher than those of OPC.

**Figure 1:**
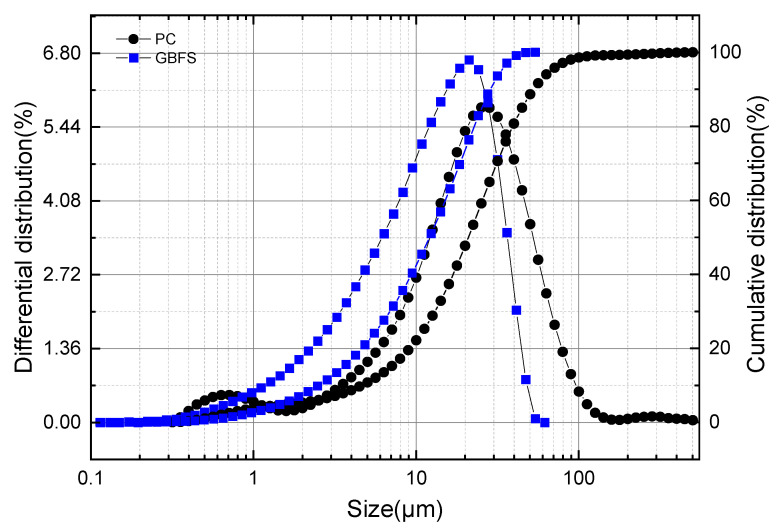
Particle size distribution of GBFS powder.

The laser particle size distribution curves of GBFS and OPC are illustrated in Figure 1. The maximum particle size of GBFS was 53.94 μm, which was significantly smaller than that of OPC (503.20 μm). The average particle size of GBFS was 12.09 μm, while that of OPC was 21.06 μm. The most probable particle sizes of GBFS and OPC were relatively close, being 21.06 μm and 26.70 μm, respectively.

**Figure 2:**
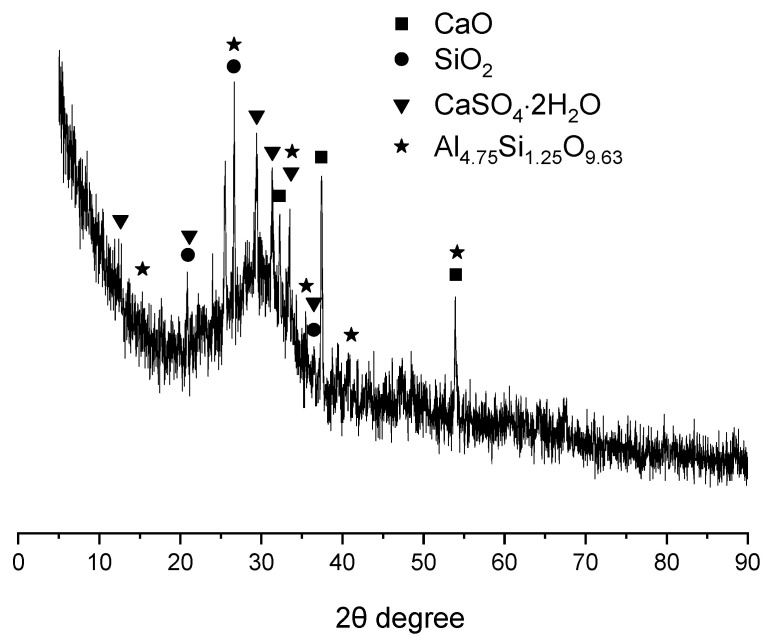
XRD pattern of GBFS.

The XRD spectrum of GBFS is presented in Figure 2. In addition to exhibiting a substantial amorphous phase content, the GBFS was found to contain crystalline phases including quartz (SiO_2_), lime (CaO), mullite (Al_4.75_Si_1.25_O_9.63_), and gypsum (CaSO_4_·2H_2_O). The presence of mullite and quartz, which are characteristic phases of fly ash (FA), suggested that a portion of FA had been incorporated into GBFS [27]. Lime and gypsum, commonly employed as activators, were likely added to enhance the reactivity of GBFS [28].

### 2.2. Setting Time and Fluidity

#### 2.2.1. Setting Time

**Figure 3:**
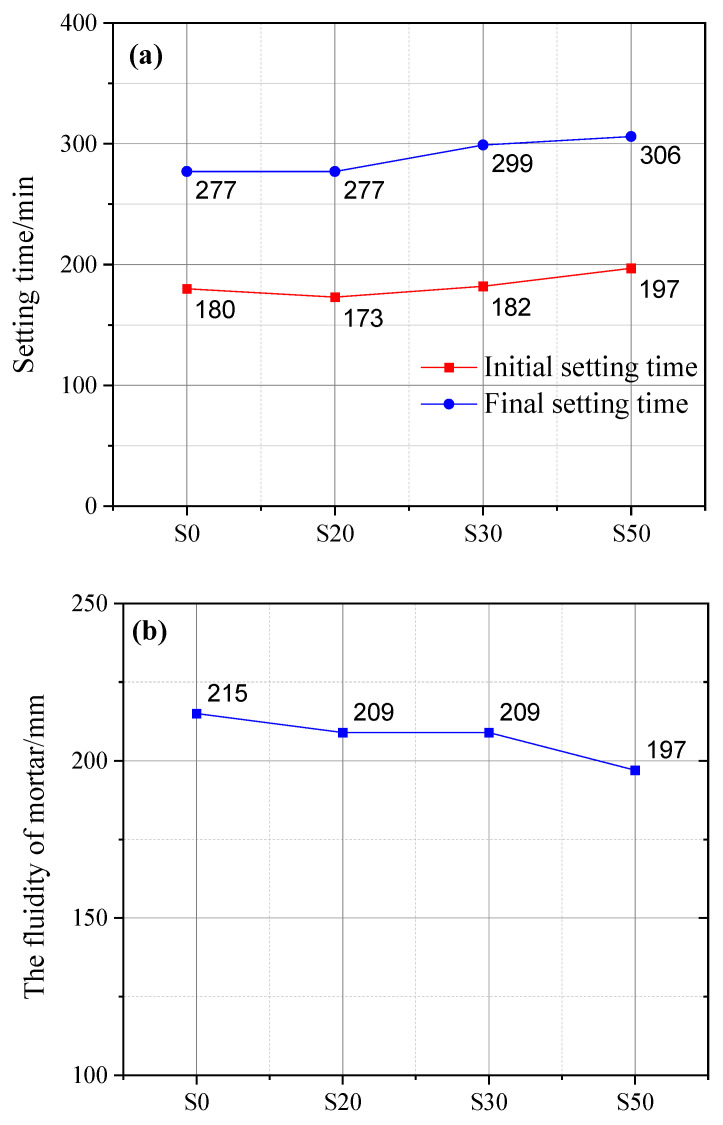
Influence of GBFS on cement setting time (**a**) and mortar fluidity (**b**).

Figure 3 illustrates the effect of GBFS on the setting time of cement paste and the fluidity of cement mortar. The initial setting time of cement paste incorporating GBFS was comparable to that of plain cement paste (PCP), which was mainly attributed to the small particle size and high pozzolanic activity of GBFS. However, a higher dosage of GBFS would prolong the initial setting time. Specifically, the initial setting time of cement paste with 50% GBFS content was prolonged by 17 min compared with that of PCP. Similarly, regarding the final setting time, the cement paste with 50% GBFS content exhibited the longest final setting time (306 min), which was 29 min longer than that of PCP, followed by the cement paste with 30% GBFS content, with an extension of 20 min. The primary cause is that an excessive GBFS dosage lowers cement clinker content, which in turn reduces hydration product yield and further extends the setting time.

It is noteworthy that the final setting time of cement paste containing 20% GBFS content was identical to that of PCP. This observation can be attributed to the accelerating effect of GBFS on cement hydration. Assuming no early-stage hydration of GBFS, the water otherwise consumed by GBFS hydration becomes fully available for cement clinker hydration. This essentially raises the effective water–cement ratio of the cement system, thereby enhancing its overall hydration extent. Furthermore, activators such as quicklime and gypsum contained in GBFS can also accelerate the reaction between OPC and GBFS [28].

#### 2.2.2. Fluidity

With an increase in GBFS content, the fluidity of mortar decreased accordingly. Specifically, when the GBFS content reached 50%, the mortar fluidity exhibited the maximum reduction of 8% compared with that of plain cement mortar (PCM). In contrast, there was no significant difference in the fluidity of mortar samples with 20% and 30% GBFS content. In addition to the gypsum and quicklime contained in GBFS [29], this phenomenon is also related to GBFS’s larger specific surface area and finer particle size [30,31,32].

At the same mass, GBFS consists of a greater number of finer particles than OPC, which significantly increases the probability of direct interparticle contact and thereby elevates the sliding resistance among solid particles. Beyond this, the fine GBFS particles can extensively adsorb free water in the fresh mortar, forming an adsorbed water film on their surfaces. As the GBFS content increases, the free water available in the mortar decreases substantially, which in turn leads to a marked reduction in the thickness of the lubricating water film on the particle surfaces. This further enhances interparticle friction, ultimately resulting in increased flow resistance and elevated viscosity of the mortar. Additionally, the fine GBFS particles are prone to agglomeration, which impedes the shear deformation induced during the mortar’s flow and consequently amplifies the mortar’s flow resistance [30,31,32].

### 2.3. Mechanical Properties

#### 2.3.1. Mortar Steam-Cured at 50 °C 

**Figure 4:**
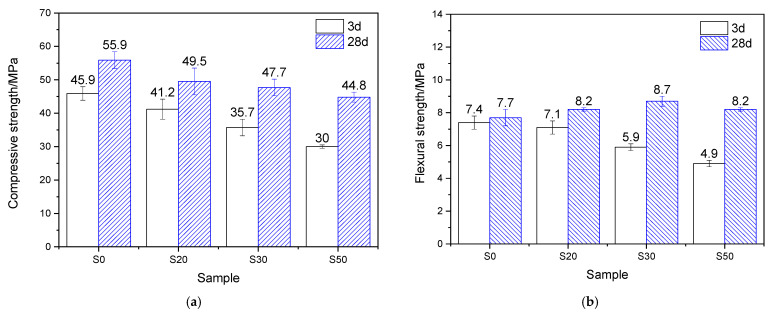
Mechanical properties of 50 °C steam-cured cement mortar: (**a**) compressive strength; (**b**) flexural strength.

The mechanical properties of the mortar, which underwent 7 h of steam-curing at 50 °C followed by standard curing to a total age of 3 days and 28 days, are presented in Figure 4. Analysis based on the strength activity index (defined as the ratio of the mechanical properties of GBFS-modified mortar to those of PCM) shows that when the GBFS content was 20%, 30%, and 50%, the compressive strength activity indices reached 89.7%, 77.8%, and 65.4%, respectively, showing a significant decreasing trend. The flexural strength activity indices were 95.9%, 79.7%, and 66.2%, respectively, which exhibited a similar variation tendency to the compressive strength activity indices but were higher than the latter.

After 28 days of curing, at GBFS contents of 20%, 30%, and 50%, the corresponding compressive strength activity indices were 88.5%, 85.3%, and 80.1%, respectively, while the flexural strength activity indices reached 106.5%, 113.0%, and 106.5%. This indicates that, compared with its effect on compressive strength, GBFS contributes more favorably to the flexural strength of mortar cured at 50 °C. This phenomenon can be attributed to the continuous hydration process: GBFS reacts with CH generated from cement hydration, forming substantial amounts of fibrous C–S–H gels, which thereby effectively reinforces the interfacial transition zone (ITZ) between the hardened cement paste and sand [10].

By comparing the mechanical properties of mortar hydrated for 3 and 28 days under 50 °C steam-curing, it is evident that GBFS content significantly regulates later-age strength development. In terms of compressive strength, the four groups of specimens exhibited increases of 21.8%, 20.1%, 33.6%, and 49.3%, respectively, demonstrating that higher GBFS content promotes greater later-stage strength gain. A similar trend was observed for flexural strength: compared with the three-day values, the flexural strength of mortar with varying GBFS contents increased by 4.0%, 15.5%, 47.5%, and 67.3%, respectively. In summary, following early-age 50 °C steam-curing, GBFS exhibits a more significant effect during subsequent curing, particularly in promoting flexural strength development.

#### 2.3.2. Mortar Steam-Cured at 80 °C 

**Figure 5:**
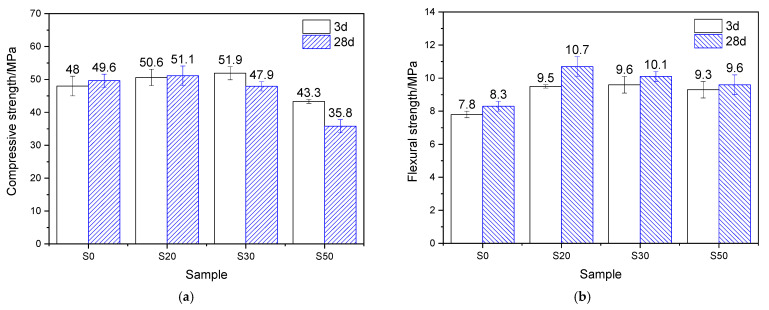
Mechanical properties of 80 °C steam-cured cement mortar: (**a**) compressive strength; (**b**) flexural strength.

The mechanical properties of the mortar, steam-cured at 80 °C for 7 h and then standard-cured to a total age of 3 days and 28 days, are shown in Figure 5. At 3 d, for groups S20, S30, and S50, the compressive strength activity indices were 105.4%, 108.1%, and 90.2%, respectively, while the flexural strength activity indices reached 121.8%, 123.1%, and 119.2%. The results show that under 80 °C steam-curing, the strength activity indices of GBFS are significantly higher than those obtained under 50 °C steam-curing, suggesting a pronounced temperature sensitivity of GBFS.

At 28 d, for groups S20, S30, and S50, the compressive strength activity indices were 103.0%, 96.6%, and 72.2%, respectively, while the flexural strength activity indices reached 128.9%, 121.7%, and 115.7%. It can be observed that the compressive strength activity indices of GBFS-modified mortar at 28 days were slightly lower than those at 3 days, whereas the flexural strength activity indices remained largely unchanged. Despite this, both the compressive and flexural strengths of group S20 at 28 days still exceeded those of the reference group S0, highlighting the beneficial role of GBFS in steam-cured precast concrete products. The behavior can be attributed to the early-stage high-temperature curing, which activated reactive components (such as Si^4+^, Al^3+^, Ca^2+^, and Mg^2+^) within the GBFS. These components subsequently reacted with the CH formed during early cement hydration, producing a considerable amount of nanoscale C–S–H gels [33]. These gels filled the pores in the mortar and thereby enhanced the development of strength [33].

Compared with the three-day mechanical property data (Figure 5), the flexural strength of all cement mortar specimens increased to varying degrees after 28 days of curing. The mortar containing 20% GBFS exhibited the highest flexural strength improvement, showing a 12.6% increase relative to its three-day counterpart at the same dosage. Plain cement mortar followed with a 6.4% rise, while Group S50 recorded the lowest growth rate, at only 3.2%. These results indicate that incorporating GBFS continues to promote the later-age flexural strength development in mortars exposed to 80 °C high-temperature curing.

Nevertheless, it should be noted that as the curing age extended, the compressive strength of mortars subjected to 80 °C steam-curing exhibited only limited improvement and even declined in some cases. For instance, plain cement mortar showed a 3.3% increase in compressive strength compared to its three-day value, while Group S20 recorded a slight increase of 1.0%. In contrast, Groups S30 and S50 exhibited reductions in compressive strength of 7.7% and 17.3%, respectively. These results suggest that the addition of GBFS is not conducive to enhancing later-stage compressive strength of mortars under 80 °C steam-curing, especially at higher GBFS dosages.

### 2.4. XRD Analysis of Hydration Products

#### 2.4.1. Mortar Steam-Cured at 50 °C

**Figure 6:**
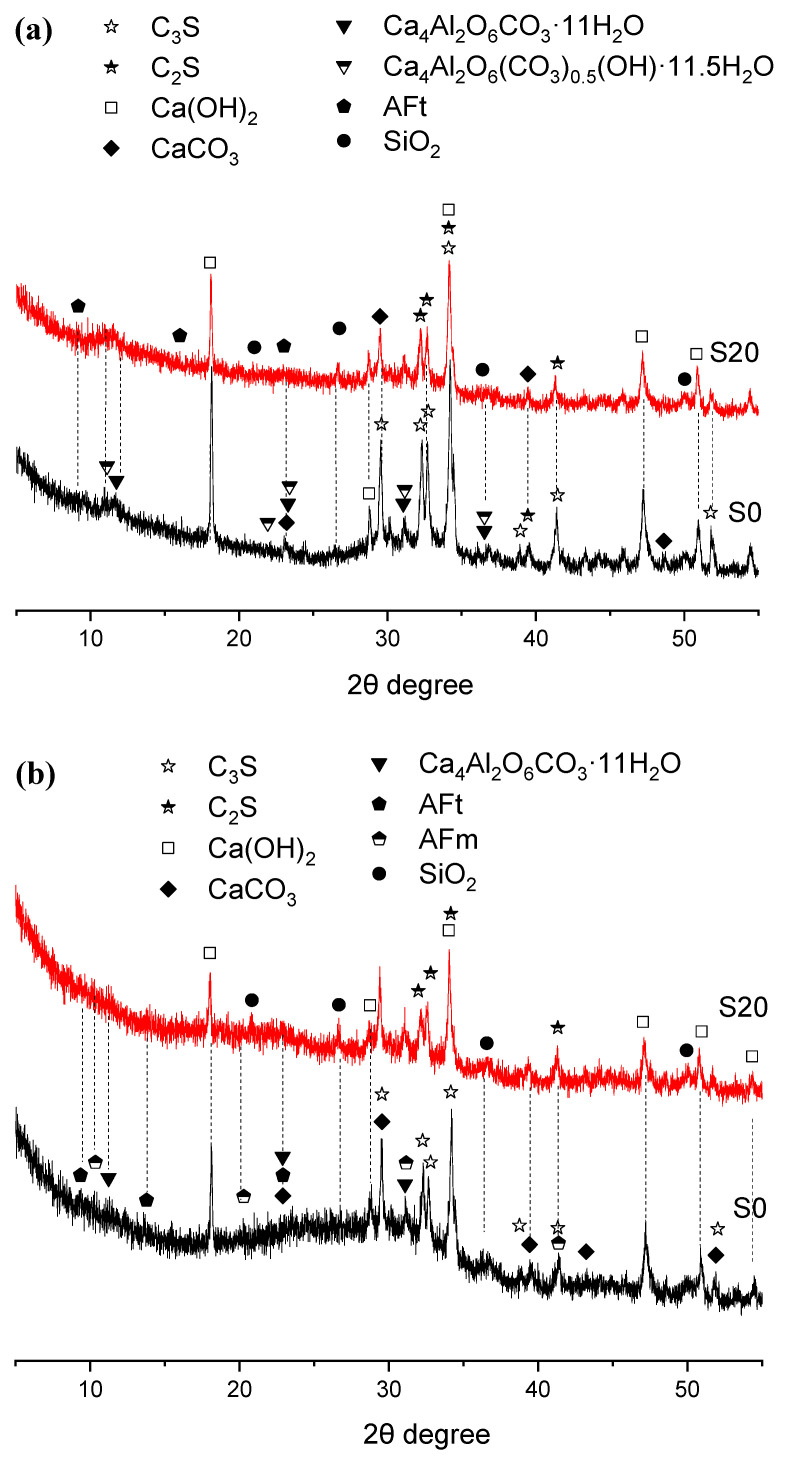
XRD patterns of hydration products in 50 °C steam-cured cement paste (**a**) 3 d; (**b**) 28 d.

**Figure 7:**
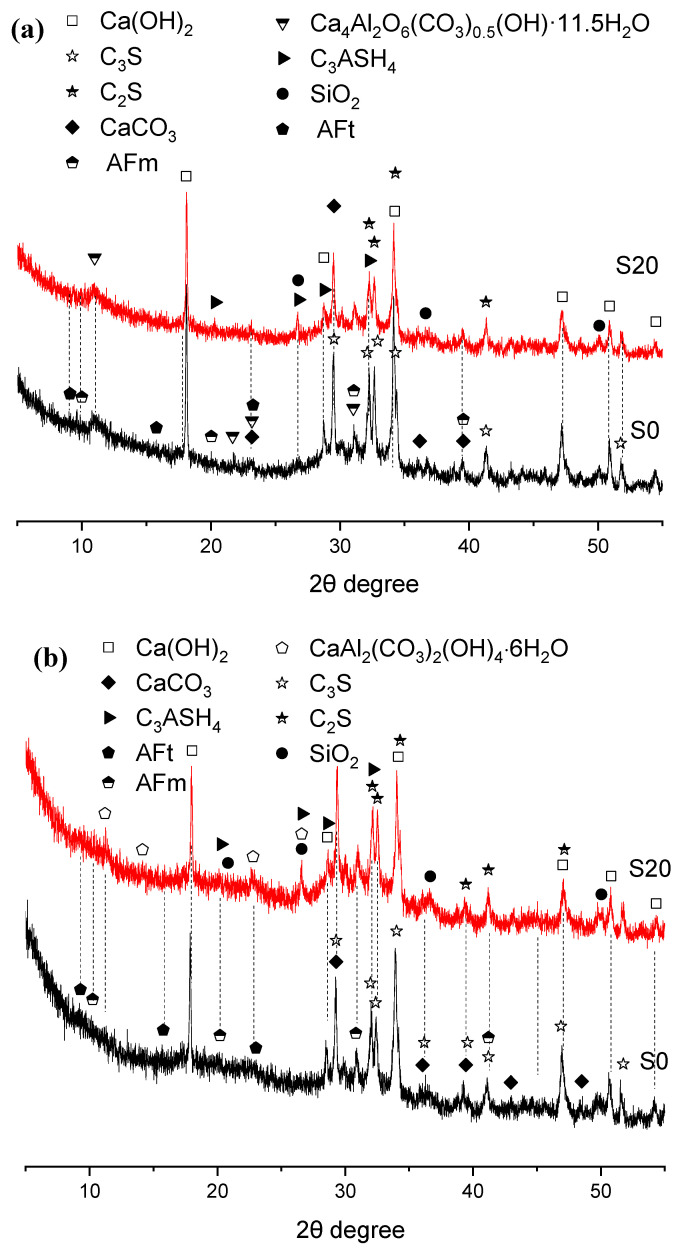
XRD patterns of hydration products in 80 °C steam-cured cement paste (**a**) 3 d; (**b**) 28 d.

To explore the mechanism underlying the effect of GBFS on the mechanical properties of steam-cured mortar, XRD analysis was performed on early steam-cured cement pastes incorporating 0% and 20% GBFS, with the results summarized in Figure 6 and Figure 7.

As shown in Figure 6, there was no significant difference in the crystalline hydration products between Groups S0 and S20 after 50 °C steam-curing. Both groups primarily consisted of calcium hydroxide (CH), calcite (CaCO_3_), ettringite (AFt), hemicarbonate hydrate (Ca_4_Al_2_O6(CO_3_)_0.5_(OH)·11.5H_2_O), and monocarbonate hydrate (Ca_4_Al_2_O_6_CO_3_·11H_2_O). The formation of monocarbonate hydrate and hemicarbonate hydrate is linked to the composition of the P·O 42.5 Portland cement used in this study, which contains about 20% supplementary cementitious materials such as fly ash (FA) and limestone powder. The limestone powder can react with C_3_A in the clinker to produce monocarbonate hydration products [34,35].

After 28 days of curing, the composition of hydration products remained largely unchanged; however, the diffraction peaks of hemicarbonate hydrates gradually diminished, accompanied by the appearance of AFm diffraction peaks.

Furthermore, the quartz (SiO_2_) detected in the XRD patterns primarily originates from FA present in the OPC and GBFS. This phase is relatively inert and shows little reactivity even under elevated temperatures.

#### 2.4.2. Mortar Steam-Cured at 80 °C

The XRD patterns of three-day hydration products of S0 and S20 cured at 80 °C under steam conditions are presented in Figure 7. The principal crystalline hydration products were calcium hydroxide, calcite, ettringite, AFm, and hemicarbonate hydrate. Evidently, elevating the steam-curing temperature from 50 °C to 80 °C favors the formation of hemicarbonate hydrate over monocarbonate hydrate, which aligns with previous findings [36].

Although temperatures exceeding 80 °C tend to induce the decomposition of ettringite, the formation of ettringite was still detected in the samples, as illustrated in Figure 7. This phenomenon can be attributed not only to the relatively high SO_3_ content in GBFS but also potentially to the subsequent water-curing applied to the specimens after high-temperature steam-curing at 80 °C, as ettringite regenerates during the post-curing period. The newly formed ettringite crystals are referred to as delayed ettringite formation (DEF) [37,38,39].

In addition, under both 50 °C and 80 °C steam-curing regimes, the diffraction peak intensity of CH crystals in S20 was significantly lower than that in S0. This observation is not only associated with the lower cement content in S20 but also indicates that elevated temperatures accelerated the hydration reaction between OPC and GBFS. GBFS can react rapidly with CH from cement hydration to form C–S–H gels and other hydration products [33]. This reaction not only fills the pores within the cement mortar, thereby improving its compactness, but also enhances the mortar’s mechanical properties, especially flexural strength, which exerts a positive effect on the development of later-age strength and durability improvement in concrete.

Furthermore, it can be observed that the incorporation of GBFS results in a more pronounced formation of hydrogarnet (C_3_ASH_4_) in the hydration products. A relatively high content of hydrogarnet is detrimental to later-stage strength development of cement concrete [40].

When the specimens were cured for 28 days, the crystalline hydration products of cement remained basically the same as those at 3 days; the main change was that the diffraction peaks of hemicarbonate hydration products gradually disappeared and were replaced by paraalumohydrocalcite (CaAl_2_(CO_3_)_2_(OH)_2_·6H_2_O). As a member of the AFm family [41], this phase transformation might be attributed to two possible reasons: first: more calcium carbonate from OPC or GBFS participated in the formation of hydrated carboaluminate with the extension of curing time; second: carbonation occurred in the specimens.

### 2.5. SEM-EDS Analysis of Hydration Products

#### 2.5.1. Mortar Steam-Cured at 50 °C

(1) 3 d

**Figure 8:**
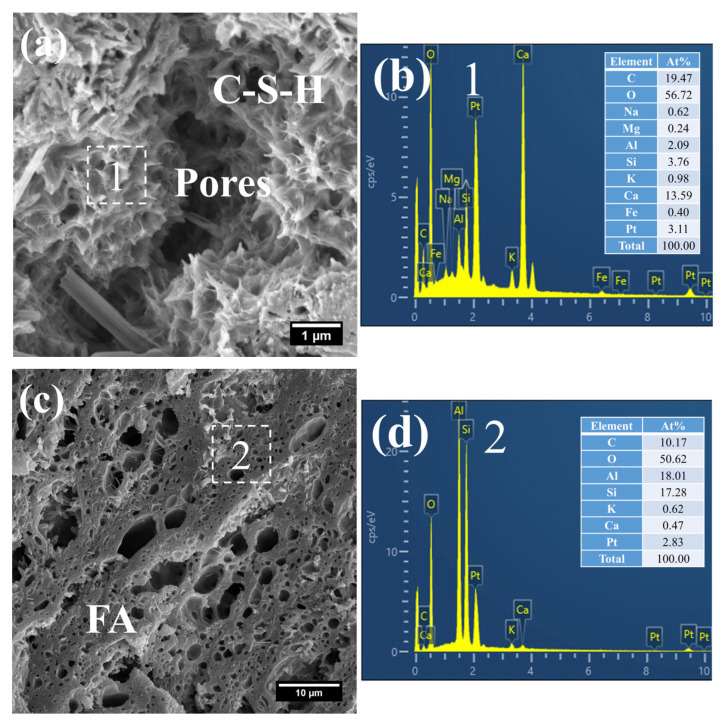
SEM-EDS images of 3 d 50 °C steam-cured S0: (**a**) honeycomb-like C–S–H; (**b**) EDS result of area 1; (**c**) cross-sectional structure of FA; (**d**) EDS result of area 2.

Figure 8 presents the morphology and composition of hydration products in 3 d S0. It can be observed that the initially fibrous C–S–H had evolved into a spheroidal and honeycomb-like structure composed of interlaced short and coarse fibers (Figure 8a). Specifically, the C–S–H phase exhibited low porosity, with its Ca/Si ratio remaining relatively high at 3.61 and the Al/Si ratio at 0.55. Even so, the presence of pores could be clearly identified in the cement paste.

In addition, a porous-structured product was observed in S0 (Figure 8c). Characterized by a relatively large particle size of approximately 50 μm, this product was primarily composed of O, Al, and Si. Based on these features, it is inferred that this product corresponds to the cross-sectional structure of crushed FA particles incorporated in OPC, a conclusion which is consistent with the aforementioned XRD analysis results.

**Figure 9:**
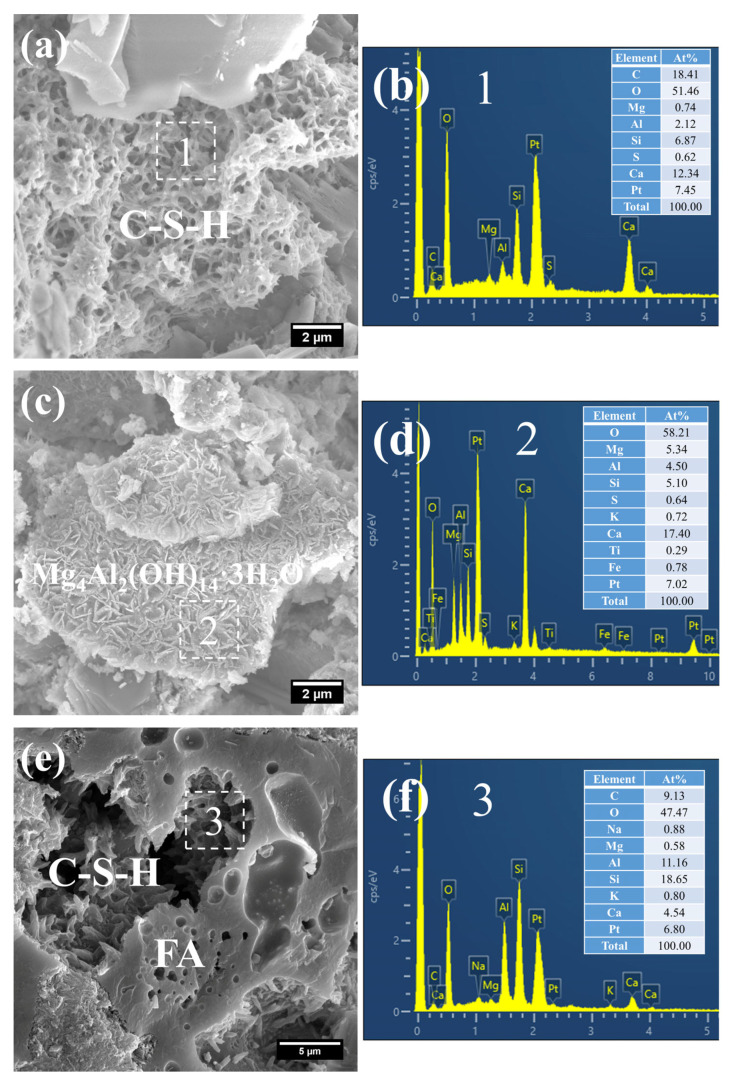
SEM-EDS images of 3 d 50 °C steam-cured S20: (**a**) mesh-like C–S–H; (**b**) EDS result of area 1; (**c**) flaky hydrotalcite; (**d**) EDS result of area 2; (**e**) C–S–H in FA; (**f**) EDS result of area 3.

The SEM-EDS images of hydration products of 3 d S20 are shown in Figure 9. Different from the morphology of C–S–H in S0, the C–S–H of S20 presented a relatively unique mesh-like structure (Figure 9a), with the mesh aperture size of approximately 500 nm. The structure was looser and more porous than that of C–S–H in the pure cement paste, indicating that the strength of S20 had further room for improvement. According to its composition (Figure 9b), the Ca/Si ratio of the C–S–H was as low as 1.80, and the Al/Si ratio was 0.31. A low Ca/Si ratio is considered the main reason for this unique mesh-like morphology of C–S–H, which indicates that GBFS affects the formation of hydration products at an early-stage.

In addition, flaky products were observed in S20 (Figure 9c). As revealed by its compositional analysis (Figure 9d), the product exhibited a relatively high Mg content, with an Mg/Al ratio of 1.19, a value close to 2. On the basis of its characteristic morphology and chemical composition, this product is inferred to be hydrotalcite.

Moreover, crushed FA particles were detected in S20 (Figure 9e). Concurrently, short, coarse fibrous C–S–H was found to form within the porous architecture of FA, with an even lower Ca/Si ratio of 0.24 (Figure 9f). This phenomenon is primarily attributed to the infiltration of a small quantity of CH into the porous structure of FA, followed by a pozzolanic reaction between the two components.

Collectively, the presence of mesh-like C–S–H, flaky hydrotalcite, and C–S–H formed within the FA matrix demonstrates that mineral admixtures including FA and GBFS in mortar cured at 50 °C had partially participated in the cement hydration reaction at this curing age.

(2) 28 d

**Figure 10:**
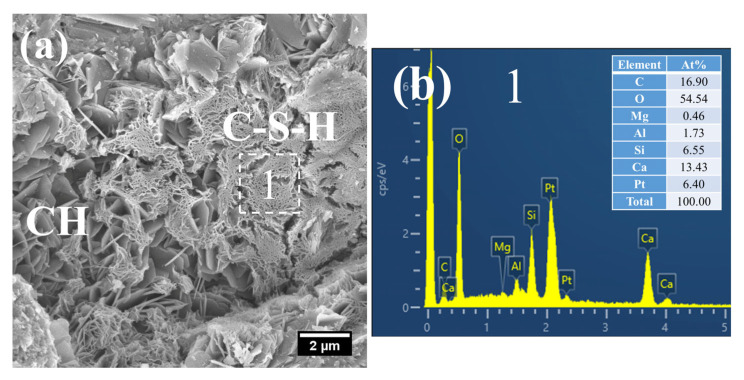
SEM-EDS images of 28 d 50 °C steam-cured S0: (**a**) dense C–S–H; (**b**) EDS result of area 1.

At 28 days of age, the C–S–H structure of S0 subjected to 7 h early-age steam-curing at 50 °C was relatively dense (Figure 10a), with a Ca/Si ratio of 2.05 and a corresponding Al/Si ratio of 0.26. In terms of both structural characteristics and chemical composition, its microstructure was comparable to that of concrete cured under standard conditions at the same age.

**Figure 11:**
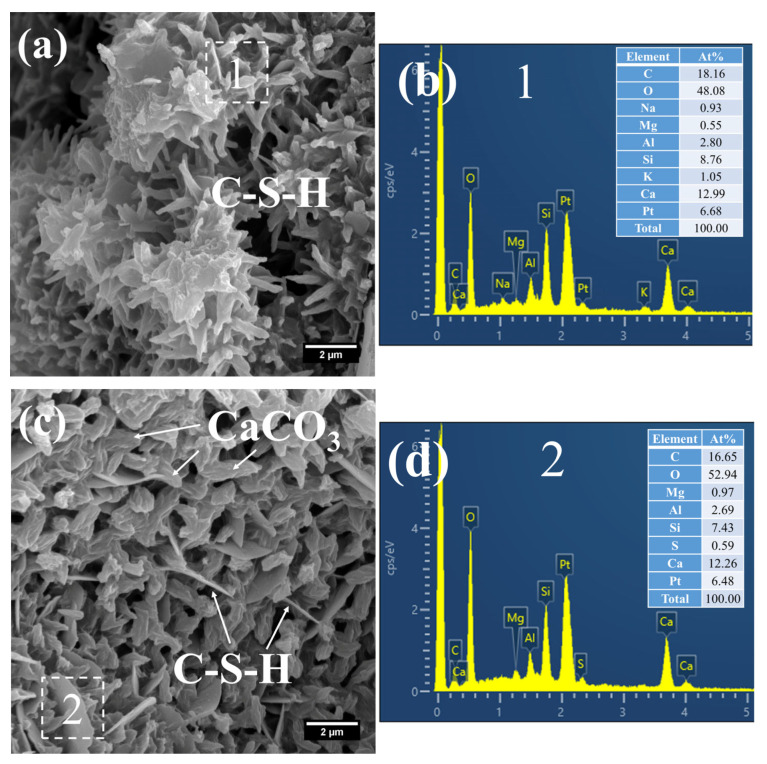
SEM-EDS images of 28 d 50 °C steam-cured S20: (**a**) fibrous C–S–H; (**b**) EDS result of area 1; (**c**) flaky C–S–H; (**d**) EDS result of area 2.

By contrast, a considerable amount of short, coarse fibrous C–S–H (Figure 11a) and flaky C–S–H (Figure 11c) were still observed in S20 under the same curing regime at 28 days. These C–S–H phases had relatively low Ca/Si ratios, registering at 1.48 (Figure 11b) and 1.65 (Figure 11d), with corresponding Al/Si ratios of 0.32 and 0.36, respectively. Notably, the short, coarse fibrous C–S–H had not yet interlaced to form a continuous network; thus, the strength of S20 is expected to further improve at later curing stages.

Furthermore, a significant amount of amorphous CaCO_3_ was detected in the hydration products at this curing age (Figure 11c). The formation of this amorphous CaCO_3_ primarily stems from the carbonation reaction of CH and C–S–H. This process not only helps mitigate the adverse effects of CH crystal growth on the ITZ but also, as amorphous CaCO_3_ is the thermodynamically least stable polymorph of CaCO_3_, its transformation into calcite can further refine the microstructure of the cementitious material [42].

#### 2.5.2. Mortar Steam-Cured at 80 °C

(1) 3 d

**Figure 12:**
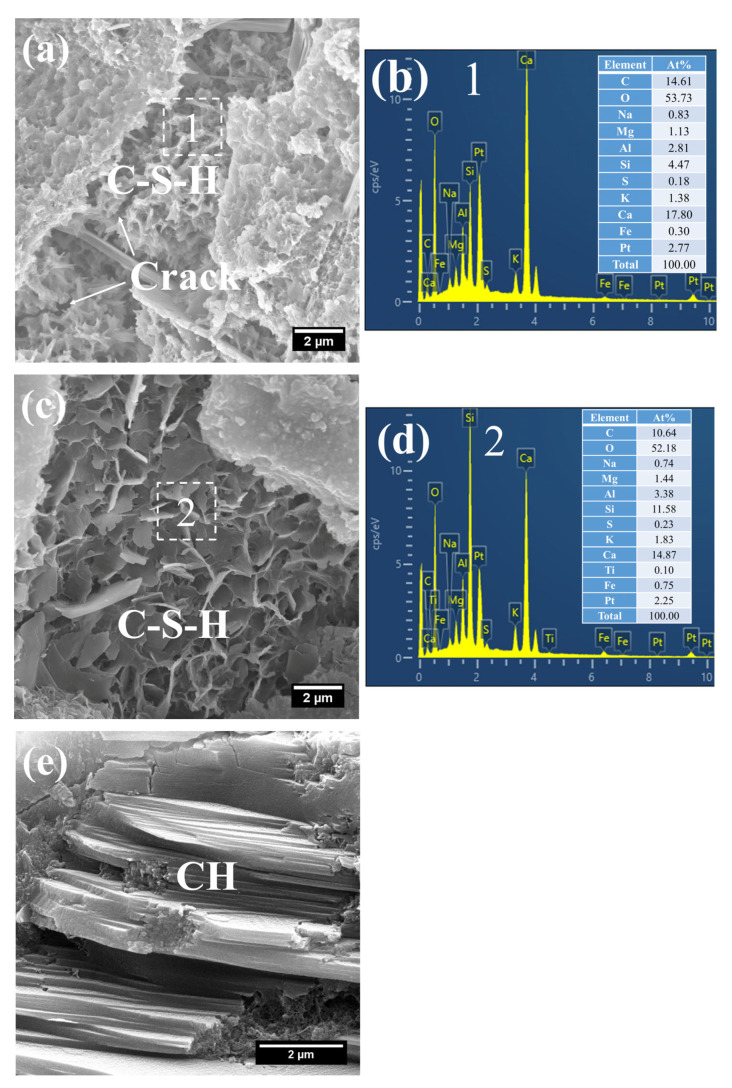
SEM-EDS images of 3 d 80 °C steam-cured S0: (**a**) honeycomb-like C–S–H; (**b**) EDS result of area 1; (**c**) foil-like C–S–H; (**d**) EDS result of area 2; (**e**) coarsely crystalline CH.

**Figure 13:**
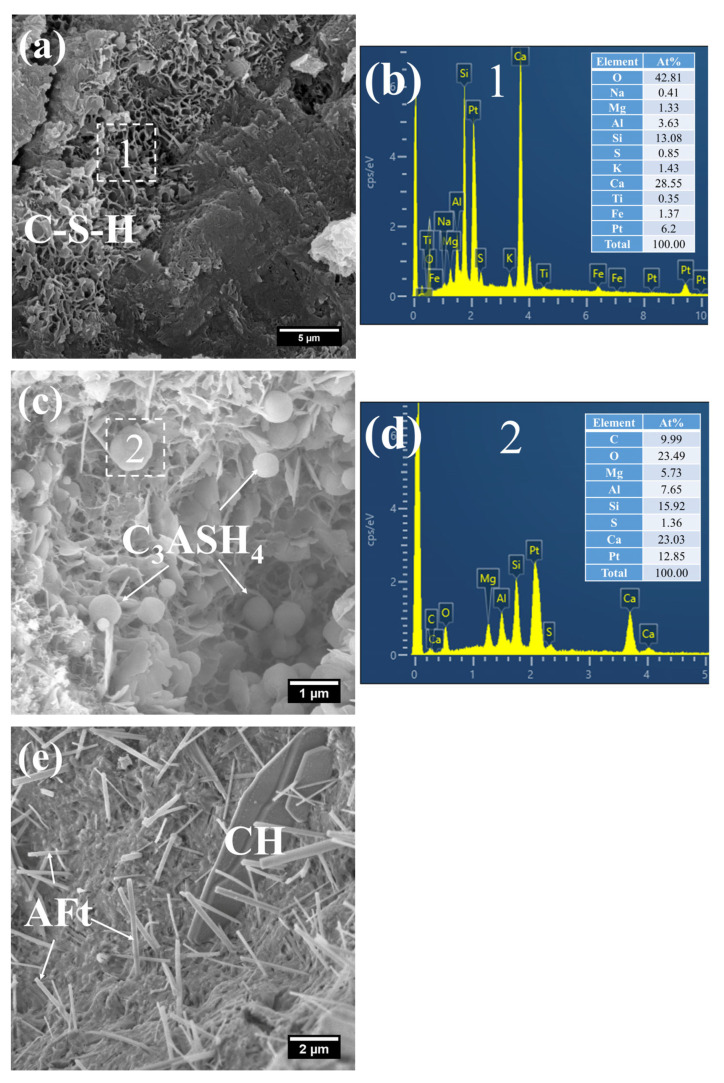
SEM-EDS images of 3 d 80 °C steam-cured S20: (**a**) honeycomb-like C–S–H; (**b**) EDS result of area 1; (**c**) spherical hydrogarnet; (**d**) EDS result of area 2; (**e**) AFt and CH.

The hydration products of the 3 d specimens subjected to steam-curing at 80 °C for 7 h are presented in Figure 12 and Figure 13.

Owing to the accelerated structural formation of the steam-cured cement paste, numerous pores and cracks remained prevalent within the hardened cement paste matrix (Figure 12a). In S0, the C–S–H phase also exhibited a honeycomb-like morphology with a relatively loose microstructure. At this curing age, its Ca/Si ratio was as high as 3.98, and the corresponding Al/Si ratio reached 0.63. Meanwhile, trace amounts of S were adsorbed on the surface of the C–S–H, indicating that the C–S–H structure remained thermodynamically unstable.

Furthermore, foil-like C–S–H was also observed in S0 (Figure 12c), with a relatively low Ca/Si ratio of 1.28 (Figure 12d). This particular C–S–H variant is inferred to be primarily the product of reactions between CH and mineral admixtures including FA. In addition, a substantial quantity of plate-like CH crystals, with relatively large particle sizes ranging from 4 to 7 μm, were detected within the cement paste (Figure 12e).

As shown in Figure 13, a considerable quantity of honeycomb-like C–S–H was observed to form in 3 d 80 °C steam-cured S20 (Figure 13a). This microstructure exhibited a relatively loose configuration, with a low Ca/Si ratio of 2.18 and a corresponding Al/Si ratio of 0.28 (Figure 13b).

Meanwhile, spherical particles were detected within the hydration products of S20 (Figure 13c), with particle sizes ranging from 0.5 to 1 μm and a measured Ca/Al ratio of 3.01 (Figure 13d). The chemical composition and morphological characteristics of these particles are consistent with those of hydrogarnet (C_3_ASH_4_); therefore, they are inferred to be hydrogarnet. This observation aligns well with the results of the aforementioned XRD analysis.

Despite exposure to 80 °C steam-curing, ettringite was still identified in S20 (Figure 13e). This phenomenon is primarily attributed to the relatively high SO_3_ content in GBFS, which is consistent with the prior XRD analysis. The ettringite is composed of two distinct fractions: one fraction forms during the steam-curing process, and the other regenerates during the subsequent water-curing stage. In addition, hexagonal plate-like CH crystals were observed in S20 (Figure 13e). Although these crystals are similar in length to those in S0, they are both smaller in overall size and less numerous.

(2) 28 d

**Figure 14:**
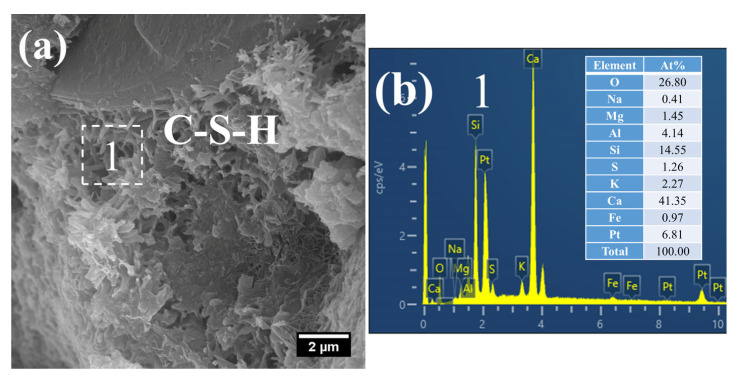
SEM-EDS images of 28 d 80 °C steam-cured S0: (**a**) loose C–S–H; (**b**) EDS result of area 1.

**Figure 15:**
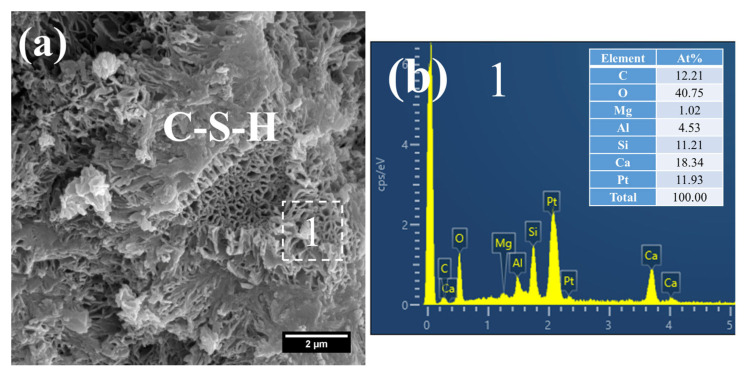

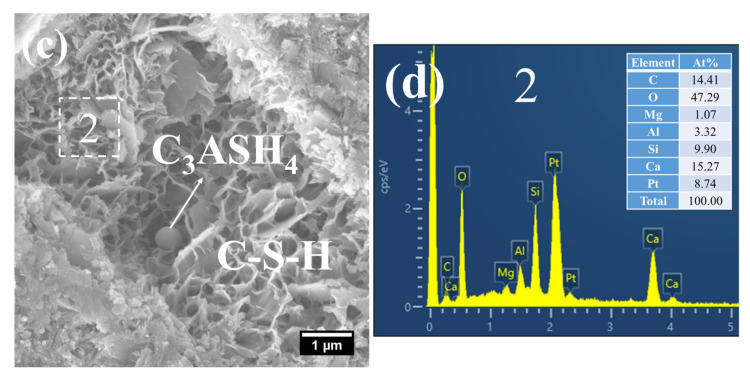
SEM-EDS images of 28 d 80 °C steam-cured S20: (**a**) honeycomb-like C–S–H; (**b**) EDS result of area 1; (**c**) hydrogarnet and honeycomb-like C–S–H; (**d**) EDS result of area 2.

At 28 d, the C–S–H structure of steam-cured S0 (80 °C) was relatively loose (Figure 14a), with a Ca/Si ratio of 2.8 and a corresponding Al/Si ratio of 0.28 (Figure 14b). By contrast, the 80 °C steam-cured S20 specimens still contained a considerable amount of honeycomb-like C–S–H at the same age, formed by the interlacing of flaky particles (Figure 15a,c). Notably, the Ca/Si ratios of this C–S–H phase were relatively low, registering at 1.64 (Figure 15b) and 1.54 (Figure 15d), with corresponding Al/Si ratios of 0.40 and 0.34, respectively. In addition, hydrogarnet was also detected in the 28 d S20 specimens (Figure 15b).

### 2.6. TG-DTG Analysis of Hydration Products

#### 2.6.1. Mortar Steam-Cured at 50 °C 

To further investigate the mechanisms underlying the effects of GBFS on mortar performance under varied steam-curing temperatures, the compositions of cement hydration products were analyzed via TG-DTG, with S0 and S20 selected as representative specimens.

**Figure 16:**
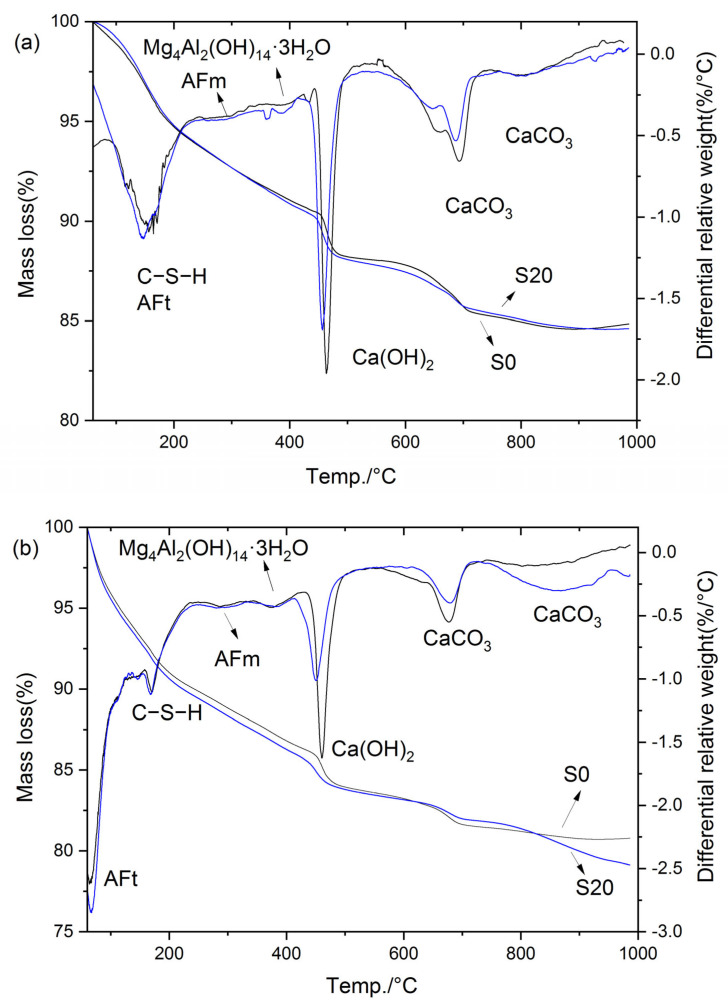
TG-DTG curves of cement paste cured at 50 °C: (**a**) 3 d; (**b**) 28 d.

Figure 16 presents the TG-DTG curves of S0 and S20 cured at 50 °C for 3 and 28 days, respectively. Specifically, as illustrated in Figure 16, the mass loss of the hardened cement paste below 80 °C was mainly attributed to the partial decomposition of ettringite, and the magnitude of this mass loss can directly reflect the ettringite content in the paste. The mass loss in the temperature range of 80 °C to 240 °C corresponded primarily to the decomposition of C–S–H, whereas the mass loss between 240 °C and 330 °C was attributable to AFm decomposition. Subsequently, the mass loss in the range of 330 °C to 410 °C was ascribed to the decomposition of hydrotalcite and hydrogarnet, while the mass loss from 410 °C to 510 °C corresponded to CH decomposition [36]. Beyond this temperature range, mass loss above 600 °C is mainly due to the decomposition of CaCO_3_. Notably, the mass loss within the temperature range of 60 °C to 600 °C reflects the quantity of cement hydration products [43]; accordingly, this mass loss is defined as the non-evaporable water content in the present study.

As indicated in Figure 16, the hydration products of the cement paste cured at 50 °C mainly consisted of C–S–H, AFt, AFm, Mg_4_Al_2_(OH)_14_·3H_2_O, Ca(OH)_2_, and CaCO_3_. Notably, the incorporation of GBFS did not alter the types of hydration products in the steam-cured cement paste. The total mass loss, mass loss in each temperature interval, CH content, and non-evaporable water content are summarized in Table 2.

It can be observed that the contents of hydration products in S0 and S20 were essentially comparable under 50 °C steam-curing. Specifically, at 3 d, the CH content of S20 was 93.3% of that of S0, the non-evaporable water content was 103.0% of that of S0, and the hydrotalcite content was 96.3% of that of S0. At 28 d, the CH content of S20 decreased to 79.6% of that of S0, the non-evaporable water content was marginally higher at 100.5% of that of S0, and the hydrotalcite content remained consistent at 96.3% of that of S0.

It is evident that early-stage steam-curing at 50 °C accelerates the pozzolanic reaction between OPC and GBFS, facilitating the formation of additional hydration products in the composite cementitious system. This contributes to the enhancement of mortar mechanical properties, resulting in S20 exhibiting mechanical performance closely comparable to that of S0. However, it should be noted that despite the higher volume of hydration products formed in S20 relative to S0, the compressive strength of S20 remains marginally lower than that of S0. This phenomenon can be attributed to the relatively high SO_3_ content in GBFS, which promoted the formation of greater quantities of ettringite in S20 (Table 2). Although ettringite is beneficial to the flexural strength of concrete, its contribution to compressive strength is considerably less significant than that of C–S–H. Furthermore, compared with the control group S0, the S20 specimen under this condition exhibited higher porosity and a smaller specific surface area in C–S–H (Figure 8, Figure 9, Figure 10 and Figure 11), which is also the main reason for its poorer mechanical performance.

Notably, the hydrotalcite contents in S20 and S0 were very close at 3 d, but the hydrotalcite content in S20 became significantly lower than that in S0 at 28 d. The disparity in hydrotalcite content between the two samples widened with the extension of curing age, which is also associated with the high SO_3_ content in GBFS. In the presence of SO_3_ and sufficient Ca(OH)_2_, the majority of Al in the cementitious system preferentially combines with SO_3_ to form ettringite or AFm [36]. This reaction consumes a large amount of Al sources available for hydrotalcite synthesis, resulting in a significantly lower hydrotalcite content in S20 than in S0. This conclusion is also verified by Figure 16b, where a greater amount of ettringite is formed in sample S20 compared with sample S0.

#### 2.6.2. Mortar Steam-Cured at 80 °C 

**Figure 17:**
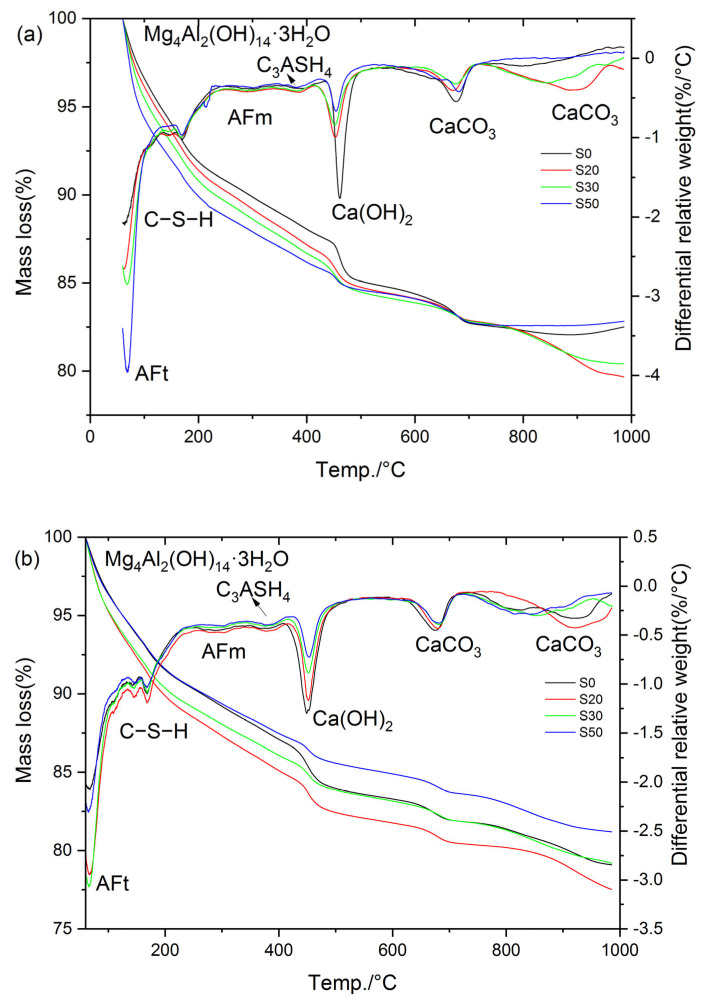
TG-DTG curves of cement paste cured at 80 °C: (**a**) 3 d; (**b**) 28 d.

Figure 17 presents the TG-DTG curves of S0, S20, S30, and S50 at 3 d and 28 d under 80 °C steam-curing conditions. In contrast to the results obtained at 50 °C, hydrogarnet was clearly detected in the hydration products of the cement paste subjected to 80 °C steam-curing. Based on the mass loss of the samples in each temperature interval illustrated in Figure 17, the mass loss of the cement paste in the corresponding temperature ranges, CH content, and non-evaporable water content were calculated. The resultant data are summarized in Table 3.

The analysis results show that under the 80 °C steam-curing condition, at 3 d, the non-evaporable water contents of S20, S30, and S50 specimens reached 101.8%, 103.1%, and 101.8% of that of S0, respectively; the contents of C–S–H were 102.9%, 104.5%, and 102.5% of those of S0, respectively; while the contents of CH were only 81.2%, 76.0%, and 42.7% of that of S0. These data characteristics indicate that early-stage high-temperature steam-curing can effectively activate the hydration reactivity of GBFS; this effect further accelerates the consumption of CH and promotes the formation of additional C–S–H gels and other hydration products.

At 28 d, the performance indexes of each specimen changed significantly: the non-evaporable water contents of S20, S30, and S50 were 109.3%, 100.7%, and 92.7% of that of S0, respectively; the C–S–H contents were 113.9%, 106.1%, and 100.0% of those of S0, respectively; and the CH contents were 89.0%, 68.5%, and 60.9% of that of S0, respectively.

At 3 d, the non-evaporable water content of S30 specimen ranked the highest among the four groups, which was the core reason for its optimal compressive strength performance. In contrast, at 28 d, the non-evaporable water content of S20 specimen was the highest, thus its compressive strength reached the peak value among the four groups. In addition, the non-evaporable water contents of the four groups of specimens at 28 days were 107.3%, 115.3%, 104.8%, and 97.7% of those at 3 days, respectively. This result fully demonstrates that when the GBFS content exceeds 20%, the growth rate of hydration products of the specimens cured by steam at 80 °C slows down significantly at later stages, which is one of the important reasons for the insufficient later-stage strength growth of S30 and S50.

Notably, the mass loss of S20, S30, and S50 in the temperature range of 330~410 °C was significantly higher than that of S0 at 3 d. This observation is exactly the opposite of the results obtained under 50 °C steam-curing, a phenomenon mainly attributed to the formation of more hydrogarnet in S20, S30, and S50 under 80 °C steam-curing conditions. Compared with OPC, GBFS contains a higher content of alumina. Under high-temperature activation, the reactivity of alumina in GBFS increases, participating more extensively in the formation of hydrated aluminates. However, these phases are unstable and gradually transform into hydrogarnet. This phase transformation process causes a reduction in solid volume, introduces pores into the concrete matrix, and consequently leads to a decrease or retrogression in concrete strength, as illustrated in Figure 5.

It should be noted that at 28 d, the mass loss of S20 and S30 specimens within the temperature range of 330–410 °C was still higher than that of the control group S0; in contrast, the mass loss of S30 and S50 specimens in the same temperature range was lower than the corresponding values of their counterparts at 3 d. This phenomenon can likely be attributed to the unfavorable effect of 80 °C high-temperature curing on the formation of ettringite. Specifically, during the 80 °C steam-curing process, most of the SO_3_ remains in the pore solution or is adsorbed onto the surface of C–S–H. Subsequently, after high-temperature curing ends and standard curing resumes, the free and unstable SO_3_ recombines with available Al sources and CH in the cement paste to form ettringite, which is a process known as delayed ettringite formation. Notably, during this delayed formation, the consumption of Al may compete with hydrotalcite, potentially leading to its partial decomposition. In contrast, hydrogarnet, due to its relatively stable structure [44], is likely to undergo minimal change throughout this process.

As can be seen from Figure 17 and Table 3, the higher the GBFS content, the greater the ettringite production of the specimens at the three-day curing age. This phenomenon is directly related to the increase in SO_3_ content in the composite cementitious material system after GBFS incorporation. At 28 d, the ettringite production in S0, S20, and S30 specimens showed a continuous growth trend, and the ettringite production in S20 and S30 remained higher than that in the control group S0; however, the ettringite production in the S50 specimens was significantly lower than the corresponding value at its three-day curing age, indicating that ettringite decomposition occurred, which was also the core reason for the negative growth of non-evaporable water content in S50.

The stability of ettringite is highly dependent on the alkalinity of the environment where it is located: ettringite can exist stably in the high-alkalinity environment of cement hydration (pH > 12.5), while it decomposes when the environmental alkalinity decreases (pH < 10.5) [45,46]. The S50 specimen had the highest GBFS content and the lowest cement content, resulting in the minimum CH content in the pore solution of the specimen and a subsequent decrease in the system pH value; thus, ettringite was more prone to decomposition. Notably, the decomposed ettringite included not only that formed at the three-day curing age but also newly generated ettringite that competed with hydrotalcite for aluminum (Al) during subsequent curing processes.

Furthermore, although the S20 specimens cured at 80 °C for 28 days generated a greater quantity of hydration products, their mechanical properties were significantly inferior to those of the same batch of S20 specimens cured at 50 °C. This phenomenon can be attributed to the tendency of elevated temperatures to induce expansion and cracking within the mortar matrix (see Figure 12a); moreover, such microcracks are incapable of being completely repaired during subsequent curing in the absence of additional remedial interventions.

### 2.7. Discussion

#### 2.7.1. Hydration Products

(1) C–S–H

As shown in Figure 8, Figure 9, Figure 10, Figure 11, Figure 12, Figure 13, Figure 14 and Figure 15, steam-curing was found to increase the early-stage Ca/Si and Al/Si ratios of C–S–H in cement pastes, with both ratios exhibiting a positive correlation with the curing temperature applied at the early-stage.

During the initial hydration period of cement, the dissolution rate of calcium ions is higher than that of silicate ions [47]. This discrepancy leads to the adsorption of a large quantity of calcium ions on the C–S–H surface, forming a calcium-enriched surface layer [48]. In addition, Kantro et al. proposed that the C–S–H formed at the early hydration stage is primarily composed of alternating C–S–H and CH layers, which inherently results in a high Ca/Si ratio of the initial C–S–H phase [49]. Elevated temperatures may further accelerate the dissolution of calcium ions, thereby leading to an even higher Ca/Si ratio in the C–S–H of steam-cured cement pastes. As hydration proceeds, the dissolution of CH and the release of C–S–H layers will subsequently drive a decrease in the Ca/Si ratio [47,48].

After the termination of steam-curing, a considerable proportion of aluminum and sulfur has been incorporated into the C–S–H phase. Specifically, most aluminum is firmly bound within the C–S–H structure via substitution of silicon atoms, while a small fraction may occupy the interlayer sites. In contrast, sulfur is loosely associated with C–S–H, predominantly through surface adsorption [37,50].

However, GBFS can effectively reduce the early Ca/Si ratio of C–S–H in steam-cured cement pastes. The primary mechanism lies in the chemical composition of GBFS: compared with OPC, GBFS contains a lower content of CaO and a higher content of SiO_2_. During the early hydration period, GBFS reacts with CH generated by cement hydration, thus lowering the concentration of calcium ions in the environment where C–S–H forms. Moreover, the higher SO_3_ content in GBFS compared with OPC enables it to compete for available aluminum ions in the cementitious system, promoting the formation of ettringite (AFt) and monosulfate (AFm) phases, thereby reducing the Al/Si ratio of C–S–H under early steam-curing conditions.

Under steam-curing conditions, the high Ca/Si ratio of C–S–H, accompanied by its relatively loose structure with wide interlayer spacing and coarse-grained CH crystallization, likely contributes to the high brittleness and low long-term strength observed in precast components. The incorporation of GBFS can effectively mitigate these drawbacks; however, attention should be paid to the potential issues induced by excessive GBFS dosage, such as the formation of hydrogarnet and delayed ettringite formation.

(2) CH

Elevated-temperature curing promotes the development of calcium hydroxide (CH) with coarse crystals (Figure 12e). CH plays multiple roles in concrete: it provides an alkaline environment to protect steel reinforcement, participates in pozzolanic reactions with mineral admixtures, and contributes partially to the strength of the hardened cement matrix. However, CH tends to crystallize around aggregate surfaces and existing hydration products, accumulating preferentially in the ITZ between aggregates and the cement paste. Since the ITZ is inherently the weakest region in concrete, the enrichment of coarse CH crystals in this area further exacerbates its vulnerability, directly impairing the overall mechanical performance of concrete. Moreover, CH is susceptible to attack by external aggressive ions, which compromises the long-term durability of concrete [51].

(3) Hydrogarnet

As can be seen from Figure 7, Figure 13, Figure 15 and Figure 17, 80 °C steam-curing condition is conducive to the formation of hydrogarnet, especially for the samples incorporated with GBFS. Hydrogarnet (C_3_AH_6_) is derived from the transformation of hydrated aluminate products including CAH10, C_2_AH_8_, and C_4_AH_13_, with the transformation rate increasing exponentially with rising temperature. This conversion process reduces the solid-phase volume of the hardened cement paste, which in turn elevates the porosity of concrete [52]. Consequently, the formation of hydrogarnet generally induces a decline in later-age strength of concrete. Owing to the extensive utilization of Si-rich mineral admixtures such as GBFS and FA, hydrogarnet typically exists in the form of C_3_ASH_4_, whose chemical formula lies in the range of C_3_AH_6_ to C_3_AS_3_ [53].

GBFS contains a relatively high content of Al_2_O_3_, and its Al_2_O_3_/SiO_2_ ratio is considerably higher than that of OPC (see Table 1). Furthermore, Al_2_O_3_ and SiO_2_ in GBFS exist predominantly in the amorphous phase. When curing temperatures exceed 80 °C, the network structure of the amorphous phase in GBFS is readily disrupted by the high-alkalinity environment of OPC, enabling it to participate rapidly in the cement hydration process. At this stage, if the cementitious system has a high content of reactive Al_2_O_3_ but a low content of reactive SiO_2_, hydration products containing Al_2_O_3_ phases in cement are dominated by hydrated aluminates. These hydrated aluminates are unstable and gradually transform into hydrogarnet, which in turn impairs concrete strength. In contrast, if the cementitious system has a low content of reactive Al_2_O_3_ but a high content of reactive SiO_2_, hydration products of the aluminous phases in cement are mainly C_2_ASH_8_, which inhibits the transformation of hydrated aluminates into hydrogarnet [54,55]. Therefore, increasing the ratio of reactive SiO_2_ to reactive Al_2_O_3_ in the cementitious system constitutes an effective approach to suppressing hydrogarnet formation.

An increase in GBFS content elevates the ratio of reactive Al_2_O_3_ to reactive SiO_2_ in the cementitious system, thereby facilitating the formation of hydrogarnet (see Table 3). It can thus be concluded that the massive formation of hydrogarnet is one of the key reasons for mechanical strength retrogression in specimens with high GBFS content.

(4) DEF

As can be seen from Figure 13 and Figure 17, the 80 °C steam-curing regime significantly promotes the formation of delayed ettringite. Research indicates that a curing regime involving high-temperature steam-curing followed by wet curing significantly promotes formation of DEF. A small amount of DEF generated in the early-stage can fill the capillary pores of the hardened cement paste, temporarily enhancing the compactness of concrete; however, this beneficial effect is only observed during the initial phase of DEF development. As ettringite crystals continue to grow via crystallization within pores and microcracks, they ultimately lead to long-term strength degradation and expansion-induced cracking in concrete [37,38,39].

Notably, delayed ettringite formation is not unique to cement systems incorporating GBFS; this hydration product is also detected in plain cement pastes, albeit at a much lower concentration. In contrast to OPC, GBFS contains elevated levels of both Al_2_O_3_ and SO_3_, two key components that provide a favorable thermodynamic environment for delayed ettringite formation (DEF). Consequently, a positive correlation is observed between GBFS dosage and the quantity of delayed ettringite generated, which is further validated by the experimental results summarized in Table 3. Therefore, to mitigate DEF, it is crucial not only to control the curing temperature but also to regulate the SO_3_ content and the ratio of SO_3_ to reactive Al_2_O_3_ within the cementitious system. In this study, higher dosages of GBFS increased the overall SO_3_ content in the cementitious system, thereby elevating the risk of DEF. It can thus be concluded that the relatively high risk of DEF induction is also one of the key causes of the long-term mechanical property degradation of specimens with high GBFS content.

(5) AFm phase

It can be observed from Figure 6 and Figure 7 that monocarbonate hydrate and hemicarbonate hydrate are produced in both plain cement pastes and GBFS-blended cement paste under steam-curing conditions. Although the formation of these two hydrates is associated with the limestone powder contained in OPC, their generation in GBFS-blended cement pastes is attributed to the synergistic effect of OPC and GBFS. Previous studies have demonstrated that hydration products such as monocarbonate hydrate and hemicarbonate hydrate are all classified as members of the AFm phase family [41]. They form when C_3_A reacts with AFt after the gypsum in the cementitious system is fully consumed, typically exhibiting a hexagonal plate-like morphology and remaining stable in concrete structures over extended periods. In AFm, sulfate ions (SO_4_^2−^) can be partially or completely substituted by hydroxyl ions (OH^−^), carbonate ions (CO_3_^2−^), and other anions; consequently, hydroxyl-bearing monosulfoaluminate hydrate (OH^−^-AFm) or carbonate-bearing monosulfoaluminate hydrate (CO_3_^2−^-AFm) is formed, and these two products are generally referred to as monocarbonate hydrate and hemicarbonate hydrate, respectively [41].

It has been well-documented in the literature that monocarbonate hydrate exhibits superior stability compared to AFm phases; it can not only stabilize the ettringite phase but also increase the solid-phase volume of the hardened cement paste at the same hydration degree, thereby reducing the porosity of the paste [34,56].

(6) Hydrotalcite

As can be seen from Figure 9, Figure 16 and Figure 17, high-temperature steam-curing promotes the formation of hydrogarnet. Hydrotalcite can immobilize detrimental ions such as chloride ions in the surrounding environment, thereby enhancing the durability of concrete [57,58,59]. It should be noted that hydrotalcite is not a phase unique to GBFS-blended hardened cement pastes but rather a group of phases commonly formed during cement hydration. In cementitious systems, the formation of hydrotalcite mainly depends on the content of reactive MgO in the matrix; since the content of reactive MgO in GBFS is significantly higher than that in ordinary cement clinker, hydrotalcite is more frequently observed in GBFS-blended cementitious materials.

#### 2.7.2. Mechanical Properties

As indicated in Figure 4 and Figure 5, the early-age reactivity of GBFS was significantly influenced by curing temperature: the higher the curing temperature, the better the early-age mechanical properties of GBFS mortar. However, later-age strength development was negatively correlated with the early-age curing temperature. The 28-day strength of GBFS mortar cured at 50 °C increased substantially compared with its 3-day strength, whereas the strength increment in mortar cured at 80 °C was relatively modest. Negative growth was even observed, particularly when the GBFS content reached 30% and 50%. The cause of this phenomenon was not only related to the formation of a large amount of hydrogarnet and delayed ettringite in the S30 and S50 specimen groups, but also closely associated with the limited increase in the amount of hydration products in the two groups of specimens at later stages (Table 3).

Although a considerable amount of hydrogarnet and delayed ettringite also formed in S20, it still exhibited the highest 28-day mechanical properties, which may be attributed to its largest total amount of hydration products (Table 3). Under the 80 °C curing condition, the performance of S20 reflects the dual effects of a low dosage of GBFS: the positive effect lies in improving the microstructure and increasing the non-evaporable water content, while the adverse effect involves inducing the formation of limited harmful phases such as hydrogarnet and delayed ettringite. In S20, the positive effect dominates. In contrast, when the GBFS replacement level exceeds the threshold of approximately 20–30%, the content of harmful phases, especially hydrogarnet and delayed ettringite, increases further, while the growth of the total amount of hydration products tends to stagnate. The resulting volumetric instability and microstructural damage then become dominant, leading to a significant decrease in strength.

Precast concrete prepared via steam-curing typically exhibits high brittleness, characterized by a low flexural strength/compressive strength ratio. The primary reasons are as follows: (1) during high-temperature steam-curing, extensive thermal damages induced by expansion and cracking are generated within the hardened cement paste (see Figure 12a); (2) high temperatures promote the extensive stacking and growth of CH, resulting in coarse CH crystals (see Figure 12e); (3) high-temperature curing significantly increases the Ca/Si ratios of early-age C–S–H. As illustrated in Figure 4 and Figure 5, the incorporation of GBFS contributes to improving the flexural strength and the flexural/compressive strength ratio of steam-cured mortar, especially when the steam-curing temperature exceeds 80 °C. The key mechanisms are as follows: (1) GBFS incorporation reduces the CH content (see Table 2 and Table 3) and the crystal size of CH in the hardened cement paste (see Figure 12e and Figure 13e); (2) it lowers the Ca/Si ratio of C–S–H; (3) the relatively high SO_3_ content in GBFS promotes the formation of additional ettringite in the mortar (see Figure 16 and Figure 17); (4) the fibrous and network-like C–S–H formed in GBFS-blended cementitious materials can bridge microcracks and enhance tensile resistance (Figure 9a, Figure 11a, Figure 13a and Figure 15a), thereby enhancing the flexural strength of the mortar. However, in a steam-curing environment exceeding 80 °C, the dosage of GBFS needs to be controlled. This is because excessive GBFS can lead to substantial formation of hydrogarnet and delayed ettringite, which adversely affects the long-term mechanical properties and durability of concrete.

#### 2.7.3. Limitations of Extending the Research Findings to Practical Precast Concrete Applications

As the research was conducted on mortar instead of concrete, direct extrapolation of the quantitative conclusions to practical precast concrete engineering applications may be subject to the following limitations:

(1) Limitations of Model Simplification

The pure mortar system lacks the skeletal effect of coarse aggregates and does not account for the complex influences of the interfacial transition zone (ITZ). Therefore, the absolute strength values and the specific degree of strength retrogression obtained from this study cannot be directly extrapolated to concrete materials.

(2) Limitations of Size Effect and Constraint Conditions

There are significant differences in thermal stress development, moisture migration, and constraint conditions between small-sized laboratory specimens and large-scale practical components. These differences may affect the extent of microcrack propagation and the hydration reaction process.

(3) Limitations of Complex Stress States

The multi-dimensional stress states borne by actual components may alter the initiation and propagation behavior of microcracks at the ITZ, thereby affecting the macroscopic mechanical properties of the material.

## 3. Conclusions

In this study, the effects of ground granulated blast-furnace slag (GBFS) on the mechanical properties and hydration products of steam-cured cement mortar were investigated, with GBFS content (0%, 20%, 30%, 50%), curing regime (50 °C for 7 h, 80 °C for 7 h), and curing age (3 d, 28 d) as the key variables. The main conclusions are drawn as follows:

(1) Increasing GBFS content prolonged the setting time and reduced mortar fluidity, although the effect was negligible at GBFS content of 20%.
(2) At 50 °C steam-curing, higher GBFS content reduced early-age strength but promoted greater later-age strength growth. However, at 80 °C steam-curing, early-age mechanical properties improved significantly. At three days, mortars with 20% and 30% GBFS even outperformed the control. However, higher-temperature curing led to limited later-age strength development and strength retrogression, especially when the GBFS content exceeded 30%.
(3) At the three-day curing age, the C–S–H gels of steam-cured mortar not only exhibited relatively high Ca/Si and Al/Si ratios but were also accompanied by calcium hydroxide crystals with a coarser grain size. However, the incorporation of GBFS was able to mitigate this phenomenon. In addition, GBFS also altered the early-age morphology of C–S–H from spherical honeycomb of short, interwoven fibers in pure cement paste to porous, flake-overlapping honeycomb structures in GBFS-modified pastes.
(4) High dosages of GBFS not only promoted the formation of hydrogarnet and delayed ettringite in mortar cured at 80 °C but also significantly slowed down the formation rate of hydration products in the corresponding specimens at later stages. These two factors jointly constitute the main reasons for the later-age strength regression of the mortar. To mitigate the formation of these undesirable phases, it is necessary to strictly regulate the curing temperature, GBFS content, and the reactive Al_2_O_3_/SiO_2_ ratio and SO_3_ level within the composite cementitious system.

## 4. Materials and Methods

### 4.1. Materials

Cement: Ordinary Portland cement (OPC) of grade P·O 42.5 was employed. It was supplied by Huai’an Conch Cement Co., Ltd (Huai’an, China) and conformed to the Chinese national standard GB 175-2023 [60]. The cement exhibited a loss on ignition of 1.6%, a specific surface area of 368 m^2^/kg, a density of 3.08 g/cm^3^, and a standard consistency water demand of 27%. The initial and final setting times were 245 min and 287 min, respectively. The 28-day compressive strength reached 57.2 MPa, and the flexural strength reached 8.8 MPa.

Granulated blast-furnace slag (GBFS): S95-grade GBFS, supplied by Jiangsu Genshen Building Materials Co., Ltd. (Huai’an, China), was used. It met the technical requirements of GB 18046-2017 [61]. The GBFS had a specific surface area of 422 m^2^/kg and a loss on ignition of 0.25%. Its compressive strength activity index—defined as the strength ratio of mortar with 50% GBFS replacement to the reference cement mortar—was 79% at 7 days and 99% at 28 days.

Sand: ISO standard sand, complying with GB/T 17671-2021 [62], was used in the mortar preparations. The sand had a fineness modulus of 2.75, an SiO_2_ content of 97%, a loss on ignition of 0.10%, and a clay content of 0.10%.

Water: Tap water, satisfying the quality requirements of the industry standard JGJ 63-2006 [63], was employed as mixing water.

### 4.2. Sample Preparation and Test Methods

#### 4.2.1. Sample Preparation

The mix proportions for this experiment are shown in Table 4. To simplify the experimental process, mortar was used to simulate concrete. The mortar was prepared with a binder-to-standard sand-to-water ratio of 450:1350:225, where the cement replacement levels with GBFS were set at 0%, 20%, 30%, and 50%, respectively. Mortar specimens were cast as 40 mm × 40 mm × 160 mm prisms. For each mix proportion, three parallel specimens were prepared to determine the compressive and flexural strengths. All specimen preparation procedures were conducted in accordance with the Chinese national standard GB/T 17671-2021 [62].

Although the curing temperature of industrial precast components typically ranges from 65 to 90 °C, the majority of relevant research have concentrated on the 50–80 °C interval [64,65,66]. More importantly, an extensive body of published literature has identified 70–75 °C as a critical threshold governing the thermal stability of ettringite [37]. Above this temperature range, ettringite is susceptible to decomposition, which can subsequently facilitate the formation of delayed ettringite (DEF) associated with potential expansion-induced damage risks. Accordingly, two curing temperatures were selected in this study: 50 °C, a sub-threshold temperature at which ettringite maintains structural stability, and 80 °C, a supra-threshold temperature at which ettringite undergoes definitive decomposition. This dual-temperature experimental setup allows for a clear, side-by-side comparison of how materials behave under two distinct curing conditions: thermally safe conditions that pose no harm and hazardous conditions that may cause thermal damage and long-term durability issues (such as delayed ettringite formation (DEF) and hydrogarnet precipitation).

**Figure 18:**
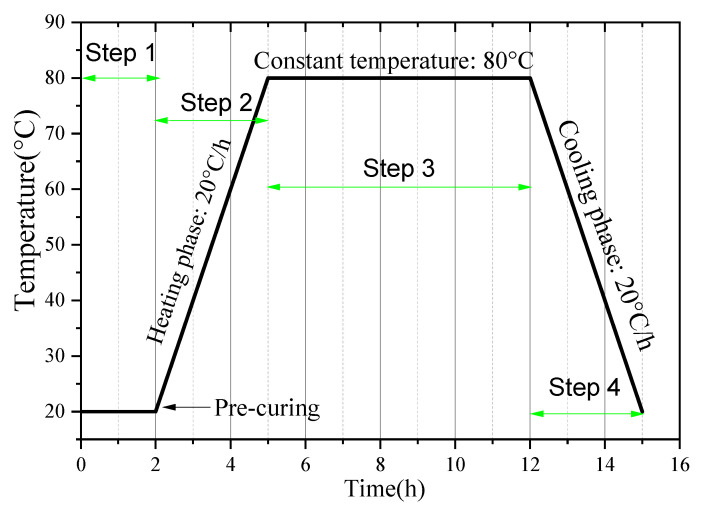
Steam-curing regime (taking 80 °C curing as an example).

The specific steam-curing regime is illustrated in Figure 18. After the mortar specimens were cast, they were placed in a standard curing chamber (20 ± 2 °C, 99% relative humidity) and allowed to rest for 2 h. The temperature was then increased at a rate of 20 °C/h until reaching the target constant temperature, which was maintained for 7 h. Subsequently, the specimens entered the cooling phase and were allowed to cool naturally to room temperature. After demolding, the specimens continued to be cured under standard conditions until reaching 3 days and 28 days, after which mechanical performance tests were conducted.

Cement pastes without sand were cast into sealed centrifuge tubes according to the proportions specified in Table 4. These pastes were cured under the same steam-curing conditions as the mortar specimens. After curing for 3 and 28 days, hydration was terminated using anhydrous ethanol. The phase composition and microstructure of the steam-cured mortar were subsequently analyzed using XRD, SEM-EDS, and TG-DTG techniques.

#### 4.2.2. Test Methods

The setting time of cement was determined in accordance with the Chinese National Standard GB/T 1346-2024 [67]. The fluidity of cement mortar was assessed following the Chinese Standard GB/T 2419-2005 [68]. The mechanical properties of mortar were evaluated based on the specifications of GB/T 17671-2021 [62].

XRD test: GBFS samples and cement paste samples with hydration terminated by anhydrous ethanol were dried in a vacuum oven at 60 °C and then ground into powder. Phase composition analysis was performed using a D8-Discover X-ray diffractometer (XRD) (Karlsruhe, Germany) with Cu Kα_1_ radiation as the radiation source, operating at a voltage of 40 kV and a current of 36 mA. The scanning rate of the X-ray diffractometer was set at 3°/min. The scanning range for GBFS was 5~90°, whereas for the hardened cement paste, the testing range was limited to 5~55°, as its primary crystalline phases are concentrated within this angular range.

SEM-EDS test: The hydrated cement paste was first broken into regular particles with a size of approximately 2~3 mm and then dried to constant weight in a vacuum drying oven at 60 °C. The samples were subsequently fixed onto the sample stage with conductive adhesive, followed by gold sputtering. The microstructure and phase composition of the hardened cement paste were then characterized using a Sirion field emission scanning electron microscope (FEI Company, Hillsboro, OR, USA) coupled with an X-ray energy dispersive spectrometer (Hillsboro, OR, USA).

TG-DTG test: For quantitative analysis of the composition of various phases in steam-cured hardened cement paste containing GBFS, thermogravimetric (TG) and derivative thermogravimetric (DTG) analyzes were conducted on the hardened paste using an STA 449 F3 Jupiter simultaneous thermal analyzer (Selb, Germany). The tests were performed under a nitrogen atmosphere at a heating rate of 20 °C/min, with the temperature ranging from 60 °C to 1000 °C.

## Figures and Tables

**Table 1:** Chemical compositions of OPC and GBFS, wt.%.

Materials	CaO	SiO_2_	Al_2_O_3_	MgO	SO_3_	MnO	TiO_2_	P_2_O_5_
OPC	63.66	22.31	5.76	1.95	1.83	2.52	0.32	0.18
GBFS	39.47	26.14	12.34	7.19	3.06	0.24	0.67	0.18

**Table 2:** Mass loss across various temperature intervals, non-evaporable water, and CH content of 50 °C steam-cured cement pastes, wt.%.

Samples	Curing Age	Total Mass Loss	<80 °C	80~240 °C	330~410 °C	CH	Non-Evaporable Water
S0	3 d	15.2	0.58	5.68	1.07	10.31	12.20
S20	3 d	15.4	0.44	5.78	1.03	9.62	12.57
S0	28 d	19.18	2.60	7.30	1.71	10.89	16.76
S20	28 d	20.82	2.91	7.45	1.40	8.67	16.84

**Table 3:** Mass loss across various temperature intervals, non-evaporable water, and CH content of 80 °C steam-cured cement pastes, wt.%.

Samples	Curing Age	Total Mass Loss	<80 °C	80~240 °C	330~410 °C	CH	Non-Evaporable Water
S0	3 d	17.44	2.09	6.92	1.64	11.18	15.58
S20		20.39	2.45	7.12	1.79	9.08	15.86
S30		19.54	2.91	7.23	1.79	8.50	16.07
S50		17.16	3.96	7.09	1.80	4.77	15.86
S0	28 d	20.86	2.29	7.17	1.67	11.87	16.72
S20		22.42	2.99	8.17	1.80	10.56	18.28
S30		20.86	3.07	7.61	1.68	8.13	16.84
S50		18.79	2.29	7.17	1.55	7.23	15.50

**Table 4:** Mix proportions of cement mortar with GBFS (g).

Sample	OPC	GBFS	Sand	Water
S0	450	0	1350 ± 5	225
S20	360	90	1350 ± 5	225
S30	315	135	1350 ± 5	225
S50	225	225	1350 ± 5	225

## Data Availability

The original contributions presented in this study are included in the article. Further inquiries can be directed to the corresponding authors.

## Abbreviations

The following abbreviations are used in this manuscript:

GBFS	Ground granulated blast-furnace slag
C–S–H	Calcium silicate hydrate
PPC	Pure Portland cement
OPC	Ordinary Portland cement
XRD	X-ray diffractometer
SEM-EDS	Scanning electron microscope coupled with an X-ray energy dispersive spectrometer
TG-DTG	Thermogravimetric and derivative thermogravimetric analyses
PCP	Plain cement paste
PCM	Plain cement mortar
CH	Calcium hydroxide
AFt	Ettringite
FA	Fly ash
AFm	Monosulfoaluminate hydrate
DEF	Delayed ettringite formation
ITZ	Interfacial transition zone
